# The Genomic Landscape of Corticotroph Tumors: From Silent Adenomas to ACTH-Secreting Carcinomas

**DOI:** 10.3390/ijms23094861

**Published:** 2022-04-27

**Authors:** Sergio Andonegui-Elguera, Gloria Silva-Román, Eduardo Peña-Martínez, Keiko Taniguchi-Ponciano, Sandra Vela-Patiño, Ilan Remba-Shapiro, Erick Gómez-Apo, Ana-Laura Espinosa-de-los-Monteros, Lesly A. Portocarrero-Ortiz, Gerardo Guinto, Sergio Moreno-Jimenez, Laura Chavez-Macias, Renata Saucedo, Lourdes Basurto-Acevedo, Blas Lopez-Felix, Carolina Gonzalez-Torres, Javier Gaytan-Cervantes, Jorge T. Ayala-Sumuano, Andres Burak-Leipuner, Daniel Marrero-Rodríguez, Moisés Mercado

**Affiliations:** 1 Unidad de Investigación Medica en Enfermedades Endocrinas, Hospital de Especialidades, Centro Medico Nacional Siglo XXI, Instituto Mexicano del Seguro Social, Ciudad de Mexico 06720, Mexico; sergenesis@gmail.com (S.A.-E.); gloriasil44@gmail.com (G.S.-R.); juaneduardomartinez300@gmail.com (E.P.-M.); keiko.taniguchi@hotmail.com (K.T.-P.); sanvela9231@gmail.com (S.V.-P.); ilanremba@gmail.com (I.R.-S.); analems@hotmail.com (A.-L.E.-d.-l.-M.); sgrenata@yahoo.com (R.S.); lbasurtoa@yahoo.com (L.B.-A.); andresburakl@gmail.com (A.B.-L.); 2 Área de Neuropatología, Servicio de Anatomía Patológica, Hospital General de México “Dr. Eduardo Liceaga”, Ciudad de Mexico 06720, Mexico; erickapo@hotmail.com (E.G.-A.); laurachm@prodigy.net.mx (L.C.-M.); 3 Instituto Nacional de Neurología y Neurocirugía “Manuel Velasco Suarez”, Ciudad de Mexico 14269, Mexico; portocarrero.lesly@gmail.com (L.A.P.-O.); radioneurocirugia@gmail.com (S.M.-J.); 4 Centro Neurológico, Centro Medico ABC, Ciudad de Mexico 01120, Mexico; gguinto@prodigy.net.mx; 5 Facultad de Medicina, Universidad Nacional Autónoma de México, Ciudad de Mexico 04360, Mexico; 6 Servicio de Neurocirugía, Hospital de Especialidades, Centro Medico Nacional Siglo XXI, Instituto Mexicano del Seguro Social, Ciudad de Mexico 06720, Mexico; belopfel@gmail.com; 7 Laboratorio de Secuenciacion, Division de Desarrollo de la Investigacion, Centro Medico Nacional Siglo XXI, Ciudad de Mexico 06720, Mexico; gonzaleztorrescaro@gmail.com (C.G.-T.); Javier_gc50@hotmail.com (J.G.-C.); 8 IDIX Biotech, Queretaro 76235, Mexico; jsumuano@gmail.com

**Keywords:** corticotroph, Cushing disease, ACTH-secreting carcinoma, single nucleotide variation, copy number variation, exome

## Abstract

Corticotroph cells give rise to aggressive and rare pituitary neoplasms comprising ACTH-producing adenomas resulting in Cushing disease (CD), clinically silent ACTH adenomas (SCA), Crooke cell adenomas (CCA) and ACTH-producing carcinomas (CA). The molecular pathogenesis of these tumors is still poorly understood. To better understand the genomic landscape of all the lesions of the corticotroph lineage, we sequenced the whole exome of three SCA, one CCA, four ACTH-secreting PA causing CD, one corticotrophinoma occurring in a CD patient who developed Nelson syndrome after adrenalectomy and one patient with an ACTH-producing CA. The ACTH-producing CA was the lesion with the highest number of single nucleotide variants (SNV) in genes such as *USP8*, *TP53, AURKA, EGFR, HSD3B1* and *CDKN1A*. The *USP8* variant was found only in the ACTH-CA and in the corticotrophinoma occurring in a patient with Nelson syndrome. In CCA, SNV in *TP53, EGFR, HSD3B1* and *CDKN1A* SNV were present. *HSD3B1* and *CDKN1A* SNVs were present in all three SCA, whereas in two of these tumors SNV in *TP53, AURKA* and *EGFR* were found. None of the analyzed tumors showed SNV in *USP48, BRAF, BRG1* or *CABLES1*. The amplification of 17q12 was found in all tumors, except for the ACTH-producing carcinoma. The four clinically functioning ACTH adenomas and the ACTH-CA shared the amplification of 10q11.22 and showed more copy-number variation (CNV) gains and single-nucleotide variations than the nonfunctioning tumors.

## 1. Introduction

The pathological spectrum of the corticotroph includes ACTH (adrenocorticotropic hormone)-secreting pituitary adenomas (PA), causing Cushing disease (CD), silent corticotroph adenomas (SCA), Crooke cell adenomas (CCA) and the rare ACTH-secreting carcinoma (ACTH-CA). Pituitary carcinomas account for 0.1 to 0.2% of all pituitary tumors and are defined by the presence of craniospinal or distant metastasis [1,2,3]. Most pituitary carcinomas are of corticotroph or lactotrope differentiation [3]. Although a few cases present initially as CA, the majority develop over the course of several months or years from apparently benign lesions [3,4]. CCA are characterized by the presence of hyaline material in more than 50% of the cells of the lesion, and most of them arise from silent corticotroph adenomas (SCA) or CD-provoking ACTH-secreting adenomas [5]. SCA are pituitary tumors with positive immunostaining for ACTH but are not associated with clinical or biochemical evidence of cortisol excess; they are frequently invasive lesions and represent up to 19% of clinically non-functioning pituitary adenomas (NFPA) [6]. ACTH-secreting PA represents up to 6% of all pituitary tumors and causes eloquent Cushing disease (CD), which is characterized by symptoms and signs of cortisol hypersecretion, including a two- to fivefold increase in mortality [7,8]. The 2017 World Health Organization (WHO) classification of PA considers not only the hormones these tumors synthesize but also the transcription factors that determine their cell lineage [9]. *TBX19* is the transcription factor responsible for the terminal differentiation of corticotrophs [9]. All tumor lesions of corticotroph differentiation are positive for both ACTH and *TBX19*.

ACTH-secreting PA causing CD are among the best genetically characterized pituitary tumors, with *USP8* somatic variants occurring in up to 25–35% of sporadic cases [9]. Yet, information regarding the molecular pathogenesis of the lesions conforming to the whole pathological spectrum of the corticotroph is scarce. The aim of the present study is to characterize the genomic landscape of pituitary tumors of corticotroph lineage. For this purpose, we performed whole exome sequencing to uncover the mutational burden (single-nucleotide variants, SNV) and copy-number variations (CNVs) of these lesions.

## 2. Results

### 2.1. Clinical and Demographic Characteristics of the Patients

A total of 10 tumor samples from 10 patients were evaluated: 4 ACTH-secreting adenomas causing clinically evident CD, three non-functioning adenomas that proved to be SCA upon immunohistochemistry (IHC), one ACTH-secreting CA with a prepontine metastasis, one rapidly growing ACTH-secreting adenoma after bilateral adrenalectomy (Nelson syndrome) in a patient with CD and one non-functioning, ACTH-producing CCA (Table 1). All except one patient were female; the mean age was 38.8 ± 16.5 years (range 17–61) (Table 1). They all harbored macroadenomas with a mean maximum diameter of 31.9 ± 13 mm (range 18–51). Cavernous sinus invasion was evident on MRI in all but one of the patients (Table 1). Homonymous hemianopia was present in seven patients, whereas right optic nerve atrophy and amaurosis were evident in patient with the ACTH-CA, and in patient with CD and pituitary apoplexy (Table 1). Detailed clinical data are included in Supplementary Table S1. Death was documented in only the patient with pituitary apoplexy, and one patient was lost during follow-up, as of October 2018.

### 2.2. General Genomic Characteristics of Neoplasms of Corticotrophic Lineage

**Figure 1:**
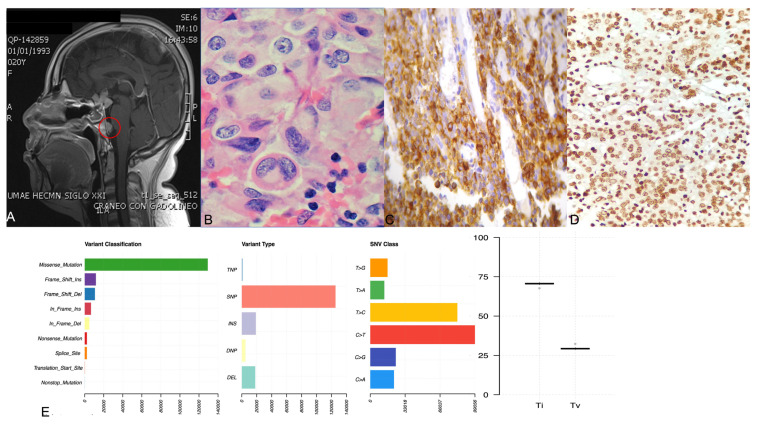
Panel (**A**) shows the gadolinium-enhanced magnetic resonance imaging of the patient with ACTH-CA, highlighting in red the metastatic lesion in the prepontine area. Panel (**B**) shows the hematoxylin and eosin staining displaying the hyaline structures in the perinuclear areas denoting a Crooke cell adenoma. Panel (**C**,**D**) depict a representative corticotroph tumor with positive ACTH and TBX19 immunohistochemistry, respectively. Panel (**E**) shows four graphics: variant classification, variant type, SNV class and transition (ti) or transversion (tv) describing the general results of exome sequencing of the corticotroph tumors.

**Figure 2:**
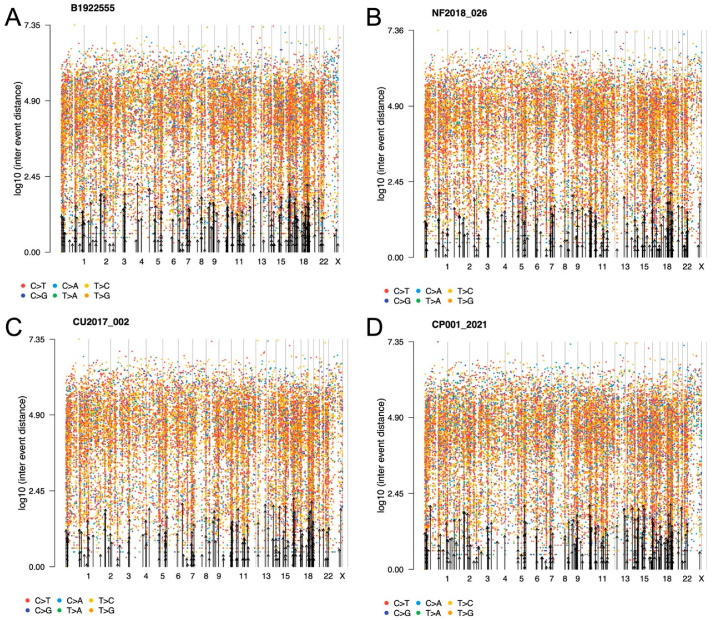
Representative rainfall plots showing the SNV alterations throughout the whole genome of corticotroph tumors (**A**) CCA, (**B**) SCA, (**C**) CD and (**D**) ACTH-CA, displaying all base changes, including transversions and transitions. No kataegis events were found. Alterations across the genome were seen in all corticotroph tumors.

Overall, approximately 18,000 variants were found, including missense, nonsense and splice-site variants as well as frameshift insertions and deletions. Of these alterations, the majority corresponded to single-nucleotide variants, followed by insertions and deletions. The three most common base changes were transitions C > T, T > C and C > G; most of the genetic changes were base transitions rather than transversions (Figure 1). There were several genes across the whole genome affected in more than one way, meaning that the same gene presented missense and nonsense variants, insertions, deletions and splice-site variants (Figure 2). Many of these variants are of unknown pathogenicity and require further investigation. Gains in genetic material were found in 44 cytogenetic regions, whereas 72 cytogenetic regions showed loss of genetic material in all corticotroph tumors.

### 2.3. ACTH-Secreting Carcinoma (Tumor 1)

**Figure 3:**
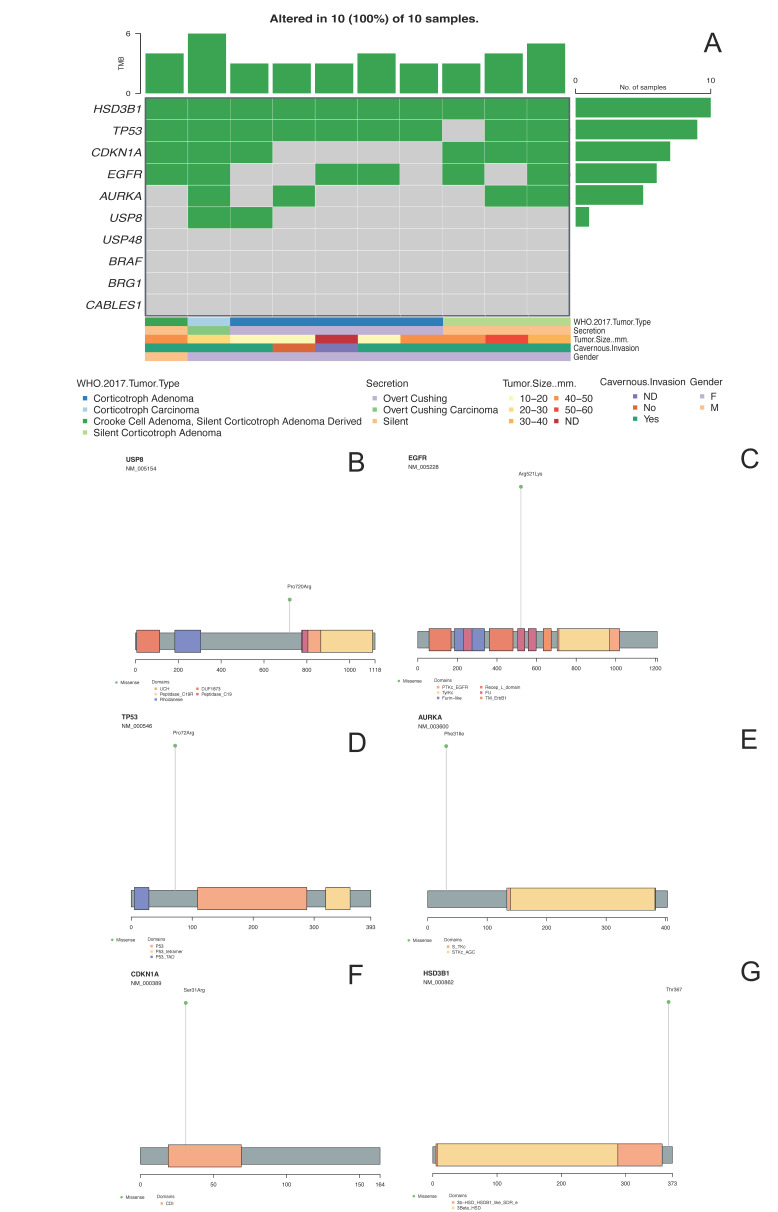
Panel (**A**) shows the oncoplot from the missense variants of the selected genes and their clinical–pathological features. Panels (**B**–**G**) depict *USP8, EGFR, TP53, AURKA*, *CDKN1A* and *HSD3B1* proteins, respectively, with the changes found in DNA impacting aminoacidic changes.

SNV missense variants were found in the genes encoding *TP53* (c.215G > C [rs1042522], p.Pro72Arg); *AURKA* (c.91T > A [rs2273535], p.Phe31Ile); *EGFR* (epidermal growth factor receptor, c.1562G > A [rs2227983], p.Arg521Lys); *HSD3B1* (3-ß-hydroxisteroid dehydrogenase, c.1100C > A [rs1047303], p.Thr367Asn); *CDKN1A* (cyclin-dependent kinase inhibitor 1A or p21, c.93C > A [rs1801270], p.Ser31Arg); and *USP8* (c.2159C > G [rs672601311], p.Pro720Arg). Interestingly, the previously reported *USP48, BRAF, BRG1* and *CABLES1* variants in pituitary CA cases were not found in this patient’s tumor (Figure 3). All SNV detected in WES experiments were validated by Sanger sequencing. The variants described were selected due to their potential pathogenic participation in other tumors and the allelic-risk association with tumorigenesis. Hereafter, all the mentioned variants in other corticotroph tumors are referred to by these aforementioned variants. Even though these same genes presented other variants, currently the significance of those variants is unknown.

**Figure 4:**
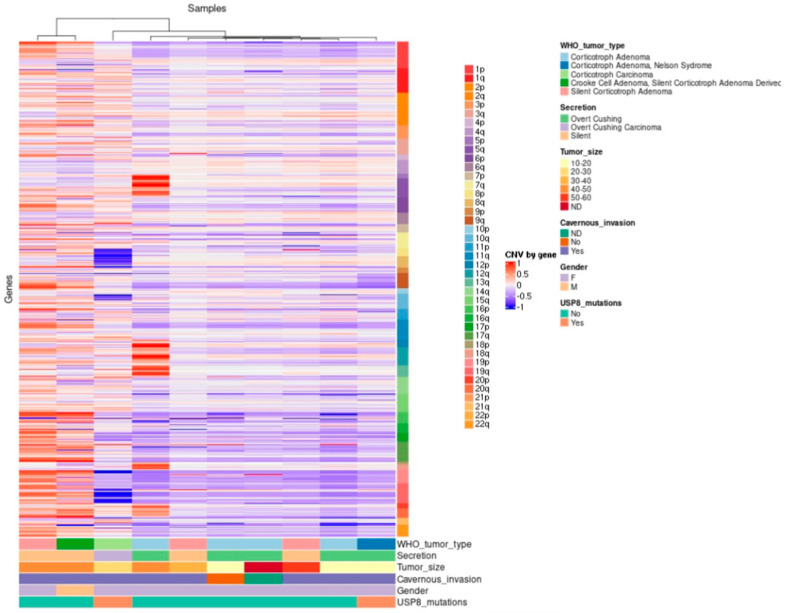
Hierarchical clustering of corticotroph tumors according to their gains and losses across the whole genome (somatic chromosomes only). High contrast was used to enhance potential CNV alterations; nevertheless, there were only 44 unique cytogenetic regions that showed gains in genetic material with statistical significance, whereas only 72 unique cytogenetic regions showed loss of genetic material with statistical significance.

In general, the pituitary CA presented more CNV alterations than the benign tumors, with 27 and 32 cytogenetic regions showing gains and losses of genetic material, respectively. The cytogenetic regions showing gains were 10q11.22, 15q11.2, 16p12.3, 1p13.2 and 20p, where genes *SYT15, POTEB, ARL6IP1, HIPK1* and *CJD6* are coded, respectively. By contrast, 8p21.2 was the cytogenetic region showing loss of genetic material. The previously reported amplification of 1p13.2 was also detected in this tumor (Figure 4) [10].

### 2.4. Crooke Cell Adenoma (Tumor 2)

The CCA showed SNV in the genes encoding *TP53, EGFR, HSD3B1* and *CDKN1A*. However, neither the genes encoding *AURKA* and *USP8* nor those encoding *USP48*, *BRAF, BRG1 and CABLES* were affected in this tumor. In CCA, only two and fifteen gains and losses were observed in copy-number variation, respectively. CNVs only showed gains in cytogenetic regions 17q12 and 10q11.22, harboring genes *CCL3L1* and *NPY4R*, respectively, whereas losses were found in cytogenetic regions 18q21.1, 15q12 and 2q11.2, harboring genes *KATNAL2, TUBGCP5* and *ANKRD36*.

### 2.5. Silent Corticotroph Adenomas (Tumors 3–5)

The three SCA shared SNVs in the genes encoding *HSD3B1* and *CDKN1A*. SCA 4 and 5 showed SNV in the genes encoding *EGFR*, whereas SNV in the genes encoding *AURKA* and *TP53* were present in SCA 3 and 5. None of the SCA were found to have SNV in the genes encoding *USP8, USP48, BRAF, BRG1* or *CABLES1*.

The SCA presented only two and eighteen gains and losses (CNV), respectively. In regard to CNV, the these clinically silent tumors presented gains of genetic material in cytogenetic regions 17q22 and 10q11.22, which harbor genes encoding *CCL3L1* and *NPY4R*. Eighteen losses were found distributed in cytogenetic regions 18q21.1, 15q12 and 2q11.2, encompassing the genes encoding *KATNAL2, TUBGCP5* and *ANKRD36*. This CNV pattern closely resembles the one found in the CCA, which is somewhat expected if we consider that both neoplasms are clinically non-functioning

### 2.6. ACTH-Secreting Adenomas (Cushing Disease) (Tumors 6–9)

SNV of the genes encoding *TP53* and *HSD3B1* were present in tumor samples from all four CD patients, whereas none of these patients harbored adenomas with SNV in the genes encoding *USP8* or *CDKN1A*. An SNV in the gene encoding *AURKA* was identified in only one of these tumors (tumor 8). *EGFR* SNV were found in tumors 7 and 9. None of the CD-causing ACTH-secreting adenomas showed the previously reported SNV in the genes encoding *USP48, BRAF, BRG1* and *CABLES1*.

CNV analysis in this group of eloquent-area corticotroph tumors revealed 25 gains and 55 losses of genetic material. The gains occurred in cytogenetic regions 17q12, 2p12, 9p24 and 10q11.22, where genes *CCL3L1, CTNNA2, FOXD4* and *NPY4R* are coded, respectively. The losses were localized in cytogenetic regions 21p12, 15q11.2, and 8p23, harboring genes *USP16*, *KLF13* and *DEF130A*, respectively. We also detected the previously reported 20p13 amplification [10].

### 2.7. ACTH-Secreting Adenoma Causing Nelson Syndrome (Tumor 10)

This patient’s tumor showed SNV in the genes encoding *USP8, TP53, HSD3B1* and *CDKN1A* but no alterations were found in the genes encoding *EGFR* and *AURKA*. This tumor and the ACTH-CA were the only two neoplasms that harbored a *USP8* variant. No SNV were identified in the genes encoding *USP48, BRAF, BRG1* and *CABLES1*. Interestingly, CNV analysis revealed the same gains and losses of genetic material found in tumors from other patients with CD.

### 2.8. Tumor Phylogenic Analysis

**Figure 5:**
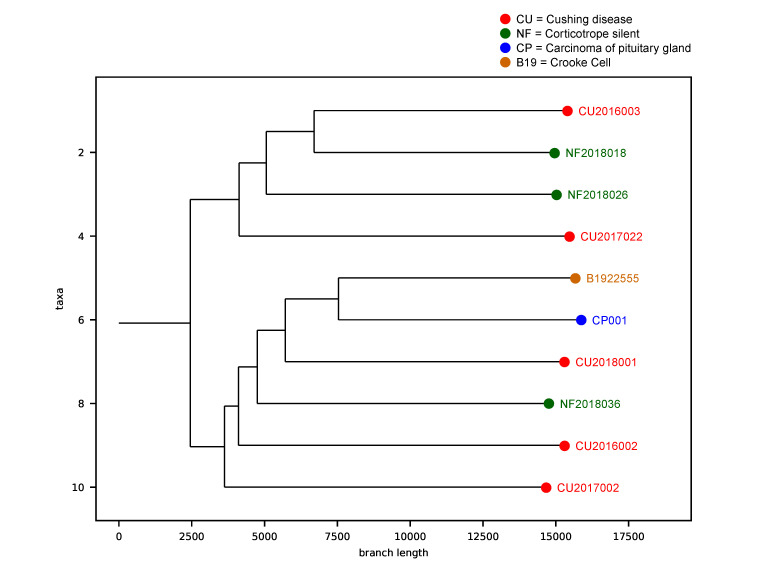
Phylogenetic analysis of the corticotroph tumors. The theoretical evolutive development of the ACTH-CA, departing from the SCA shows two main clades. The first clade, characterized by *ATF7IP* gene, comprises 2 of the 3 SCA and 2 of the 5 ACTH-adenomas causing CD. The second clade is characterized by the gene encoding *MSH3* and includes the CCA, the ACTH-CA, one of the 3 SCA and 3 of the 5 most aggressive ACTH adenomas causing CD, including the adenoma of the patient with Nelson syndrome. Red dots represent the Cushing Disease provoking adenomas, green dots represent the silent corticotroph tumors, brown dot represent the Crooke cell adenoma and the blue dot represent the corticotroph carcinoma.

We performed a phylogenetic inference analysis to unravel a hypothetical sequential step transformation from an SCA to a functioning ACTH-secreting adenoma and finally to an ACTH-CA. The theoretical evolutive development of the ACTH CA, departing from the SCA, shows two main clades, with the smallest one comprising two of the three SCA and two of the five ACTH-adenomas causing CD. Since these four tumors have the same SNV profile, we can assume that they harbor the genes that must be altered to make possible the transition from a silent to a clinically eloquent adenoma; the gene encoding *ATF7IP* (c.1589A > G [rs3213764], p.K529R) characterizes this clade. The second and largest clade includes the CCA, the ACTH-CA, one of the three SCA and three of the five most aggressive ACTH adenomas causing CD, including the adenoma of the patient with Nelson syndrome. This clade represents the molecular alterations required to evolve from a CD-causing ACTH-adenoma to a more aggressive tumor, or even to a CA and is characterized by the gene encoding *MSH3* (c.235A > G [rs1650697], p.I79V) (Figure 5).

### 2.9. Correlation between Gene Variants and Clinicopathological Features

The *USP8* variant positively correlated with increased tumor mass (*p = 0.019*). The *CDKN1A* variant was significantly associated with silent tumors (*p = 0.036).* The rest of the genetic variants did not correlate with any of the clinicopathological features tested. The presence of the *EGFR* variant was not distinctly associated with any of the clinical parameters and was equally present in functional as well as non-functional tumors (*p = 0.392)*. *AURKA* SNV did not correlate with any of the features, including recurrence (*p = 0.524)*. Detailed statistical results are presented in Supplementary Table S2.

## 3. Discussion

Corticotrophs are highly specialized cells of the anterior pituitary that synthesize and secrete hormones that are essential for the maintenance of homeostasis. In this study, we sequenced the exome of 10 corticotroph tumors, including three SCA, four ACTH adenomas causing CD, an ACTH adenoma in a patient with Nelson syndrome, a CCA and an ACTH-CA in total, representing the broad pathological spectrum of this cell. Our results portray the genomic landscape of all the neoplasms that are known to affect the corticotroph.

The neoplasm with the highest number of genomic abnormalities, including SNV and CNV, was the ACTH-CA, followed by the CCA and the CD tissues. Of all the genes harboring SNVs, six were found to be present in at least two of our tumor samples: *HSD3B1, TP53, CDKN1A, EGFR, AURKA* and *USP8*.

The *HSD3B1* gene encodes a rate-limiting enzyme required for all pathways of dihydrotestosterone synthesis and is abundantly expressed in adrenal tumors. Gain of function of this *HSD3B1* variant, which has a global allelic prevalence of 0.69678 [11], results in resistance to proteasomal degradation with the consequent accumulation of the enzyme and has been associated with a poor prognosis in patients with prostate cancer [12]. Nine of the ten corticotroph tumors in our cohort harbored an SNV of the tumor suppressor gene *TP53*. The *TP53* variant described in our cohort has been reported to be present in 80% of non-functioning pituitary adenomas and is apparently associated with a younger age at presentation and with cavernous sinus invasion [13]. Furthermore, this *TP53* variant results in a reduced expression of *CDKN1A* and an increased expression of vascular endothelial growth factor (*VEGF*) as well as an increased cellular proliferation rate [13]. *CDKN1A* (also known as p21) is a cyclin-dependent kinase inhibitor regulating cell cycle progression. The SNV described in our study was reported to alter DNA binding ability and expression and has a global allelic frequency of 0.086945 [14]. This cyclin-dependent kinase inhibitor SNV was found to be associated with breast carcinoma [15] and lung cancer [16]. The presence of this SNV has not been previously explored in pituitary adenomas, although *CDKN1A* is downregulated in clinically non-functioning pituitary adenomas of gonadotrophic lineage but not in hormone-secreting tumors [17]. *EGFR* encodes a transmembrane tyrosine kinase receptor, activation of which leads to mitogenic signaling [18]. This gene is upregulated in several cancers and represents a target for molecular therapies [19]. The *EGFR* SNV described in our corticotroph tumor series was found to be associated with the response to neoadjuvant chemotherapy in patients with breast and lung cancer [18]. *EGFR* is normally expressed in corticotrophs, where it participates in the regulation of *POMC* (proopiomelanocortin) gene transcription and cellular proliferation [20]. The *EGFR* rs2227983 has a 0.264334 global allelic frequency [21]. *AURKA* is a cell-cycle regulatory serine/threonine kinase that promotes cell cycle progression by the establishment of the mitotic spindle and centrosome separation [22]. Alterations of these gene are related to centrosomal amplification, dysfunction of cytokinesis and aneuploidy [22]; it has a global allelic frequency of 0.18078 [23]. This same SNV has been associated with overall cancer risk, particularly breast, gastric, colorectal, liver and endometrial carcinomas, but it has never been formally studied in pituitary tumors [22]. Activating somatic variants of the gene encoding *USP8* were recently found in 25–40% of ACTH-secreting adenomas causing CD [24,25]. Patients harboring these variants are usually younger, more frequently females and were found to have higher long-term recurrence rates in some but not all studies [26,27]. *USP8* mediates the deubiquitination of *EGFR* by inhibiting its interaction with protein *14-3-3,* which in turn prevents its proteosomal degradation. Signaling through the recycled deubiquitinated *EGFR* is increased, leading to increased *POMC* transcription and cellular proliferation. Most activating *USP8* variants are located within its *14-3-3* binding motif [24,25]. Recently, *USP8* and *TP53* SNV were described in corticotroph tumors as drivers of aggressive lesions [28]. To our knowledge, *USP8* variants have not been evaluated in patients with pituitary carcinomas, and none of the previously mentioned studies have included patients with Nelson syndrome. In our cohort, neither the CCA nor the SCA showed variants in *USP8*, in concordance with previously published studies [25,29], or in the genes *USP48, BRAF, BRG1* and *CABLES1* [9], and none of them were present in our cohort.

Genetic structural variations in the human genome can be present in many forms, from SNV to large chromosomal aberrance [30]. CNV are structurally variant regions, including unbalanced deletions, duplications and amplifications of DNA segments ranging from a dozen to several hundred base pairs, in which copy-number differences have been observed between two or more genomes [31,32]. CNV are involved in the development and progression of many tumors and occur frequently in PA [30,33]. Hormone-secreting pituitary tumors show more CNV than non-functioning tumors [34]. Accordingly, our non-functioning SCA and CCA had considerably fewer chromosomal gains and losses than the CD-causing adenomas and the ACTH-CA. Expectedly, the ACTH-CA had significantly more cytogenetic abnormalities than any other tumor in our series. Interestingly, the ACTH-adenomas causing CD, the SCA and the CCA shared the gain of genetic material in 17q12, highlighting their benign nature. The 17q12 amplification has been described in gastric neoplasms [35]. The only cytogenetic abnormality shared by all types of corticotroph tumors was the gain of genetic material in 10q11.22. Amplification of 10q11.22 was previously described in Li–Fraumeni cancer predisposition syndrome [36]. The ACTH-CA, the CCA and one SCA clustered together showing a related CNV pattern; this CNV profile could be reflective of the aggressive nature of these neoplasms, since both CCA and SCA can follow a clinically aggressive course [5,6].

Our results show that all lesions conforming to the pathological spectrum of the corticotroph share some of the SNV and CNV profiles. These genomic changes are consistent with the potential existence of a continuum, whereby silent tumors can transform into a clinically eloquent tumor and finally to carcinoma, or at least a more aggressive tumor. It can also be interpreted as the common SNV shared by aggressive tumors. It is known that silent corticotroph adenomas may switch into a hormone-secreting tumor [37] and are considered a marker for aggressiveness and a risk factor for malignancy since most of the carcinomas are derived from functioning hormone-secreting adenomas. Our phylogenetic inference analysis showed that the genes *ATF7IP* and *MSH3* could participate in a tumor transition ending in aggressive entities or even carcinomas. *ATF7IP* is a multifunctional nuclear protein mediating heterochromatin formation and gene regulation in several contexts [38], while *MSH3* is a mismatch-repair gene [39]. Events related to heterochromatin remodeling and maintenance have been related to aggressive pituitary adenomas and carcinomas [40]. Additionally, alterations in mismatch-repair genes are related to pituitary tumor aggressiveness and resistance to pharmacologic treatment [41,42]. The variants described in *ATF7IP* and *MSH3* are related to prostate and colorectal cancer, respectively [43,44]. There is evidence suggesting that the *ATF7IP* variant could be deleterious because it leads to a negative regulation of transcription [45]. Thus, these events could be biologically relevant to corticotroph tumorigenesis, although more research is needed.

## 4. Conclusions

We have shown genomic evidence that within the tumoral spectrum of the corticotroph, functioning ACTH-secreting lesions harbor more SNV and CNV than non-functioning ACTH adenomas. The ACTH-secreting CA shows more genomic abnormalities than the other lesions, underscoring its more aggressive biological behavior. Phylogenetic inference analysis of our data reveals that silent corticotroph lesions may transform into functioning tumors, or at least potentially, into more aggressive lesions. Alterations in genes *ATF7IP* and *MSH3,* related to heterochromatin formation and mismatch repair, could be important in corticotroph tumorigenesis. The main drawback of our study is the limited sample size. We are currently increasing the number of samples to corroborate our findings and to be able to perform a more comprehensive complementary phylogenetic analysis of our data. Finally, further research is needed to uncover the roles of these variants in corticotroph tumorigenesis.

## 5. Materials and Methods

### 5.1. Patients and Tumor Tissue Samples

Ten pituitary tissues were collected: one ACTH-CA, one CCA, three SCA, and five ACTH-secreting PA causing CD, including the tumor of a patient who developed Nelson syndrome after bilateral adrenalectomy. All tumors included in the study were sporadic and were collected from patients diagnosed, treated and followed at the Endocrinology Service and the Neurosurgical department of Hospital de Especialidades, Centro Médico Nacional Siglo XXI of the Instituto Mexicano del Seguro Social, Hospital General de Mexico “Dr. Eduardo Liceaga” and Instituto Nacional de Neurologia y Neurocirugia “Manuel Velazquez”. All participating patients were recruited with signed informed consent and ethical approval from the Comisión Nacional de Ética e Investigación Científica of the Instituto Mexicano del Seguro Social, in accordance with the Helsinki declaration. 

CD was diagnosed according to our standard protocol. Briefly, the presence of hypercortisolism was documented based on two screening tests, namely a 24 h urinary free-cortisol level above 130 µg and the lack of suppression of morning (7:00–8:00) cortisol after administration of 1 mg dexamethasone the night before (23:00) to less than 1.8 µg/dL, followed by a normal or elevated plasma ACTH to ascertain ACTH-dependence. Finally, an overnight, high-dose (8 mg) dexamethasone test, considered indicative of a pituitary source, and a cortisol suppression > 69%, provided that a pituitary adenoma was clearly present on magnetic resonance imaging (MRI) of the sellar region. In none of the 10 patients included in the study was inferior petrosal venous sampling necessary to confirm the pituitary origin of the ACTH excess. Invasiveness was defined by the presence of tumor within the cavernous sinuses (CS).

DNA was extracted from paraffin-embedded tumor tissues using the QIAamp DNA FFPE tissue kit. From frozen tumors, DNA was obtained using the Proteinase K-ammonium acetate protocol.

### 5.2. Construction and Sequencing of Whole Exome Libraries

Exome libraries were prepared according to the Agilent SureSelect XT HS Human All exon v7 instructions. Briefly, 200 ng of DNA was enzymatically fragmented with Agilent SureSelect Enzymatic Fragmentation Kit. Fragmented DNA was end-repaired and dA-tail was added at DNA ends; then, molecular barcode adaptors were added, followed by AMPure XP bead purification. The adaptor-ligated library was amplified by PCR and purified by AMPure XP beads. DNA libraries were hybridized with targeting exon probes and purified with streptavidin-coated magnetic beads. The retrieved libraries were amplified by PCR and purified by AMPure XP beads and pooled for sequencing in NextSeq 500 using Illumina flow cell High Output 300 cycles chemistry. All quality controls of the libraries were carried out using Screen tape assays and quantified by Qubit fluorometer. Quality parameters included a DNA integrity number above 8 and a 100X sequencing depth aimed with at least 85% of coverage.

### 5.3. Bioinformatics Analysis

The fastq files were subjected to quality control using FastQC v0.11.9, the adapters were removed using Cutadapt v3.4, the alignment was carried out with Burrows–Wheeler Alignment Tool v0.7.17 with the -M option to ensure compatibility with Picard and GRCh38 as a reference genome. The marking of duplicates as well as the sorting was carried out with Picard v2.26.4 with the AddOrReplaceReadGroups programs with the option SORT_ORDER = coordinate and MarkDuplicates, respectively. Variant calling was carried out using Genomic Analysis Toolkit (GATK) v4.2.2.0 following the Best Practices guide (available at https://gatk.broadinstitute.org/) [46] and with the parameters used by Genomic Data Commons (GDC), available at https://docs.gdc.cancer.gov/ [47]. The GATK tools used were CollectSequencingArtifactMetrics, GetPileupSummaries, CalculateContamination and Mutect2. Mutect2 was run with the latest filtering recommendations, including a Panel of Normal and a Germline Reference from the GATK database. Filtering was performed with the CalculateContamination, LearnReadOrientationModel and FilterMutectCalls tools with the default parameters. For the calculation of CNV GISTIC v2.0.23 was used with the parameters used by GDC. Catalog of Somatic Mutation in Cancer (COSMIC) was used to uncover pathogenic variants. For the analysis of variants and CNV, the maftool v2.10.0 and ComplexHeatmap 2.10.0 packages were used. All analyses were carried out on the GNU/Linux operating system under Ubuntu v20.01.3 or using the R v4.0.2 language in Rstudio v2021.09.0+351. A second bioinformatics pipeline was also used, SureCall software (Agilent) with the default parameters used for SNV variant calling. The variants found by both algorithms were taken as reliable SNV. Data were deposited in Sequence Read Archive hosted by National Center for Biotechnology Information under accession number PRJNA806516.

Phylogenetic tree inference (PTI) was run by means of the default parameters using matrices for each sample. These matrices contain an identifier for each variant, mutant read counts, counts of reference reads and the gene associated with the variant. The only PTI parameter was Allele Frequency of Mutation and was used to improve the speed of the algorithm. Briefly, PTI uses an iterative process on the variants shared between the samples. First, it builds the base of the tree using the variants shared by all the samples; second, it eliminates these variants and establishes a split node; and third, it eliminates the variants of the sample that produced the division (split). PTI iteratively performs these three steps for all division possibilities. Each tree is given a score based on an aggregated variant count, and the tree with the highest score is chosen as the optimal tree.

### 5.4. Sanger Sequencing forConfirmation of Exome Findings

Exome variant findings in exome sequencing were validated by Sanger sequencing using BigDye Terminator v3.1 Cycle Sequencing kit (ThermoFischer) in a 3500 Genetic Analyzer. Primers used for *USP8* [48], *TP53* [49], *EGFR* [50], *AURKA* [51], *CDKN1A* [52,53] and *HSD3B1* sequencing have been previously reported.

### 5.5. Hormone and Transcription Factor Immunohistochemistry

Paraffin-embedded, formalin-fixed tissue blocks were stained with hematoxylin–eosin and reviewed by a pathologist. Tumors were represented with a 2-fold redundancy. Sections (3 μm) were cut and placed onto coated slides. Immunostaining was performed by means of the HiDef detection HRP polymer system (Cell Marque, CA, USA), using specific antibodies against each pituitary hormone (TSH, GH, PRL, FSH, LH and ACTH) and the lineage-specific transcription factors TBX19, POU1F1 and NR5A1, as previously described [54]. Two independent observers performed assessment of hormones and transcription factors expression at different times.

### 5.6. Statistical Analysis

Two-tailed Fisher exact tests and Student’s *t* tests were used to evaluate the relationship between the identified gene variants and clinicopathological features. A *p* value of <0.05 was considered statistically significant. Statistical software consisted of SPSS v28.0.1

## Figures and Tables

**Table 1:** Clinical features of the tumors analyzed and SNV present in each tumor.

Tumor Number	Age/Sex	Clinical Diagnosis	Pathological Diagnosis	Max. Tumor Diameter (mm)	Cav. Sinus Invasion	Vision Abnormal	Pituitary Surgery	SNV
1	28/F	Cushing disease	ACTH-Carcinoma	28	Yes	Yes	4 TSS, 1 TCS	*USP8, TP53, AURKA, EGFR, HSD3B1, CDKN1A*
2	52/M	Non-Functioning	Crooke Cell Adenoma	44	Yes	Yes	1 TSS	*TP53, EGFR, HSD3B1, CDKN1A*
3	61/F	Non-Functioning	Silent ACTH-Adenoma	51	Yes	Yes	2 TSS	*CDKN1A, HSD3B1, AURKA, TP53*
4	45/F	Non-Functioning	Silent ACTH-Adenoma	45	Yes	Yes	2 TSS, 1 TCS	*CDKN1A, EGFR, HSD3B1*
5	52/F	Non-Functioning	Silent ACTH-Adenoma	31	Yes	Yes	1 TSS	*CDKN1A, AURKA, TP53, EGFR, HSD3B1*
6	54/F	Cushing Disease	ACTH-Adenoma	44	Yes	Yes	1 TCS	*HSD3B1, TP53,*
7	40/F	Cushing Disease	ACTH-Adenoma	18	Yes	No	1 TSS	*EGFR, TP53, HSDB3B1*
8	18/F	Cushing Disease	ACTH-Adenoma	18	No	No	2 TSS	*HSD3B1, TP53, AURKA,*
9	17/F	Cushing Disease	ACTH-Adenoma	23	Yes	Yes	1 TCS	*HSD3B1, TP53, EGFR*
10	21/F	Nelson Syndrome	ACTH-Adenoma	17	Yes	Yes	2 TSS	*USP8, TP53, HSD3B1, CDKN1A*

## Data Availability

Data were deposited in Sequence Read Archive hosted by National Center for Biotechnology Information under accession number PRJNA806516.

## Supplementary Materials

The following supporting information can be downloaded at: https://www.mdpi.com/article/10.3390/ijms23094861/s1.
