# Supplementary material for: The Genomic Landscape of Corticotroph Tumors: From Silent Adenomas to ACTH-Secreting Carcinomas
Source: Int J Mol Sci. 2022 Apr 27;23(9):4861. doi: 10.3390/ijms23094861 (PMC9106092; doi:10.3390/ijms23094861)
Supplement: Supplementary file 1 [file ijms-23-04861-s001.zip › Supplementary Table S1.pdf]

Clinical diagnosis	Pathological diagnosis	Macro*[2] /Micro	Max. tumoral diameter (mm)	Cav. Sinus invasion	Visual abnorm.	Preoperative Medical Tx	ACTH (pg/mL) Preop/Postop	CORTISOL (µg/dL) Preop/Postop	UFC (µg/24h) Preop/Postop	DST (µg/dL) Preop/Postop	Biochemical /Tumoral Remission	Mortality
Cushing Dis	ACTH-PA	Macro	44	Yes	Yes	None	217/X	48.39/40.17	1428/496	11.5/X	No/No	LFU
Cushing Dis	ACTH-PA	Macro	18	Yes	No	None	68/X	22.85/17.95	242/299	12.8/0.611	No/No	No
Cushing Dis	ACTH-PA	Macro	18	No	No	None	98.8/53.5	15.4/10.1	978.8/67.8	13.5/3.9	No/No	No
Cushing Dis	ACTH-PA	Macro	23	Yes	No	None	22.9/X	X/X	245/X	28.2/X	No/No	Yes
Nelson Syn	ACTH-PA	Macro	17	Yes	Yes	RTx	1386/833*[3]	X/X	298/59.8*[4]	26.3/X*[4]	Yes/No	No
Cushing Dis	ACTH-CA	Macro	28	Yes	Yes	RTx, TMZ, KTZ	60/50	30/30	1200/X	X/X	No/No	No
Non Funct	SCA	Macro	51	Yes	Yes	None	10.4/X	X/1.15	X/X	X/X	NA/No	No
Non Funct	SCA	Macro	45	Yes	Yes	None	X/X	11/11.49	X/X	X/X	NA/No	No
Non Funct	SCA	Macro	31	Yes	Yes	None	41.77/X	6.64/10.08	X/X	X/X	NA/Yes	No
Non Funct	CCA	Macro	44	Yes	Yes	None	X/X	5.98/X	X/X	X/X	NA/No	LFU

F: Female, M: Male, TCS: Transcranial surgery, TSS: Transsphenoidal surgery, Sx: Surgery, UFC: Urinary Free Cortisol, DST: 1mg Dexamethasone suppression test, LFU: Lost to follow up, X: no data, TMZ: Temozolomide, KTZ: Ketoconazole, RTx: Radiotherapy, NA: not applicable

*[1] Age at diagnosis

*[2] Macro was defined as >10mm in any tumoral diameter

*[3] after adrenalectomy; before and after 2nd TSS

*[4] prior to adrenalectomy; before and after 1st TSS
