# Supplementary material for: The Genomic Landscape of Corticotroph Tumors: From Silent Adenomas to ACTH-Secreting Carcinomas
Source: Int J Mol Sci. 2022 Apr 27;23(9):4861. doi: 10.3390/ijms23094861 (PMC9106092; doi:10.3390/ijms23094861)
Supplement: Supplementary file 1 [file ijms-23-04861-s001.zip › Supplementary Table S2.pdf]

Stastisstical analysis from the SNVs in the coritcotroph tumors

UPS8

	WT	Variant	p
**WHO 2017 Tumor Type**<br>**Silent Corticotroph**<br>**Corticotroph Adenoma**	 4<br>4	 0<br>2	 <br>0.467
**Secretion**<br>**Overt Cushing carcinoma**<br>**Overt Cushing**<br>**Silent**	 0<br>4<br>4	 1<br>1<br>0	 0.082
**Tumor Size (mm) 1**	33 ± 12	38 ± 19	**0.019***
**Tumor Size (mm) 2**	38 ± 19	20 ± 14	0.267
**Cavernous Invasion (yes)**	7	2	1.00
**Gender (female)**	7	2	1.00
**Recurrence (yes)**	4	1	1.00
**CNV**<br>Neutral/Loss<br>High/Gain	 5<br>3	 1<br>1	1.00

AURKA

	WT	Variant	p
**WHO 2017 Tumor Type**<br>**Silent Corticotroph**<br>**Corticotroph Adenoma**	 2<br>4	 2<br>2	 <br>1.00
**Secretion**<br>**Overt Cushing carcinoma**<br>**Overt Cushing**<br>**Silent**	 0<br>4<br>2	 1<br>1<br>2	 <br>0.287
**Tumor Size (mm) 1**	30 ± 11	30 ± 14	1.00
**Tumor Size (mm) 2**	33 ± 20	35 ± 21	0.903
**Cavernous Invasion (yes)**	6	3	0.40
**Gender (female)**	5	4	1.00
**Recurrence (yes)**	2	3	0.524
**CNV**<br>Neutral/Loss<br>High/Gain	 3<br>3	 3<br>1	0.571

TP53

	WT	Variant	p
**WHO 2017 Tumor Type**<br>**Silent Corticotroph**<br>**Corticotroph Adenoma**	 1<br>0	 3<br>6	 <br>0.400
**Secretion**<br>**Overt Cushing carcinoma**<br>**Overt Cushing**<br>**Silent**	 0<br>0<br>1	 1<br>5<br>3	 <br>0.435
**Tumor Size (mm) 1**	40 ± 0	29 ± 12	0.393
**Tumor Size (mm) 2**	50 ± 0	32 ± 19	0.406
**Cavernous Invasion (yes)**	1	8	1.00
**Gender (female)**	1	8	1.00

**Recurrence (yes)**	1	4	1.00
**CNV**<br>Neutral/Loss<br>High/Gain	 0<br>1	 6<br>3	0.400

HSD3B1 = no hay diferencias porque es una constante (todo esta mutado)

EGFR

	WT	Variant	p
**WHO 2017 Tumor Type**<br>**Silent Corticotroph**<br>**Corticotroph Adenoma**	 1<br>3	 3<br>3	 <br>0.571
**Secretion**<br>**Overt Cushing carcinoma**<br>**Overt Cushing**<br>**Silent**	 0<br>3<br>1	 1<br>2<br>3	 <br>0.392
**Tumor Size (mm) 1**	33 ± 15	28 ± 10	0.646
**Tumor Size (mm) 2**	33 ± 26	35 ± 15	0.871
**Cavernous Invasion (yes)**	3	6	0.400
**Gender (female)**	4	5	1.00
**Recurrence (yes)**	2	3	1.00
**CNV**<br>Neutral/Loss<br>High/Gain	 3<br>1	 3<br>3	0.571

CDKN1A

	WT	Variant	p
**WHO 2017 Tumor Type**<br>**Silent Corticotroph**<br>**Corticotroph Adenoma**	 0<br>4	 4<br>2	 <br>0.076
**Secretion**<br>**Overt Cushing carcinoma**<br>**Overt Cushing**<br>**Silent**	 0<br>4<br>0	 1<br>1<br>4	 <br>**0.036***
**Tumor Size (mm) 1**	25 ± 10	33 ± 12	0.272
**Tumor Size (mm) 2**	25 ± 20	40 ± 18	0.258
**Cavernous Invasion (yes)**	3	6	0.400
**Gender (female)**	4	5	1.00
**Recurrence (yes)**	2	3	1.00
**CNV**<br>Neutral/Loss<br>High/Gain	 3<br>1	 3<br>3	0.571
